# Supplementary material for: Hair of the Dog: Identification of a Cis-Regulatory Module Predicted to Influence Canine Coat Composition
Source: Genes (Basel). 2019 Apr 26;10(5):323. doi: 10.3390/genes10050323 (PMC6562840; doi:10.3390/genes10050323)
Supplement: Supplementary file 1 [file genes-10-00323-s001.zip › Supplemental Materials.docx]

**Supplemental figure and table legends**

**Figure S1. Quantile-quantile (Q-Q) plots for SNP and WGS GWAS.**

Q-Q plots for expected (x-axis) versus observed (y-axis) -log_10_ p-values are shown for each GWAS. Genomic inflation values were calculated from GEMMA-derived association results of single- versus double-coated dogs in both the Illumina SNP (A) and whole genome sequence (B) GWAS. Lambda values for both are close to the optimal 1.

**Figure S2. Covariate analysis correcting for additional hair phenotypes.**

(A) The original SNP GWAS, the same data as Figure 1A, is reproduced here for comparison.

We assigned previously published hair phenotypes per breed and included them as covariates to correct for three additional features: furnishings (B), hair length (C), shedding potential (D), and all three features combined (E). In all associations, the single SNP on CFA28 (arrows) remains and is often the most associated.

**Table S1. Individuals and phenotypes used in the Illumina SNP array**

All of the dogs (n=1116) used in the Illumina SNP array GWAS are listed with their coat number as defined by breed standard (2=double; 1=single), hair length (1 – 5), furnishings (1=presence), and shedding potential (0 – 1).

**Table S2. Individuals and phenotypes in the WGS GWAS**

All of the dogs (n=237) used in the whole genome association are listed. Phenotypes are given only for coat number; covariate analysis was not performed on WGS data. Genome sequencing coverage is listed separately for the combined autosomes and chromosome X; all dogs had >10x coverage on the autosomes. Individuals (n=40) used for structural variant analysis have “Yes” designation in the “SV” column.

**Table S3. Significant SNPs from the Illumina SNP array GWAS**

All significant SNPs (p<5x10^-8^; n=9) from the Illumina SNP association are listed with their allele frequencies for both coat phenotypes. The CFA28 locus is the most significant association.

**Table S4. Significant SNPs from the WGS GWAS**

All significant SNPs (n=87) from the whole genome sequencing association are listed. The CFA28 locus is the most significant association. Thirteen (13) variants lie on CFA1 which was found to be associated with other hair phenotypes. Seventy-four (74) variants are found on CFA28. Each variant is listed with its derived allele frequency for the ancestral wolves, double-, and single-coated dogs. For CFA28 variants, genomic positions, nearest gene product, and the significance level they exceed are all listed. All variants in linkage disequilibrium with the most associated variant (chr28:24,863,224) are listed in the column “LD Region.”

**Table S5. Structural variant genotypes in double- and single-coated WGS**

Structural variant caller outputs for both DELLY and CNVnator for CFA28. Genomic position, variant type, and individual genotypes are listed. We identify variants that are within our LD region (“ROI” column) as well as plus/minus one (1) megabase on either side (“ROI+1MB” column). No structural variants segregate between two phenotypes.
