# Supplementary figures and images for: Hair of the Dog: Identification of a Cis-Regulatory Module Predicted to Influence Canine Coat Composition
Source: Genes (Basel). 2019 Apr 26;10(5):323. doi: 10.3390/genes10050323 (PMC6562840; doi:10.3390/genes10050323)

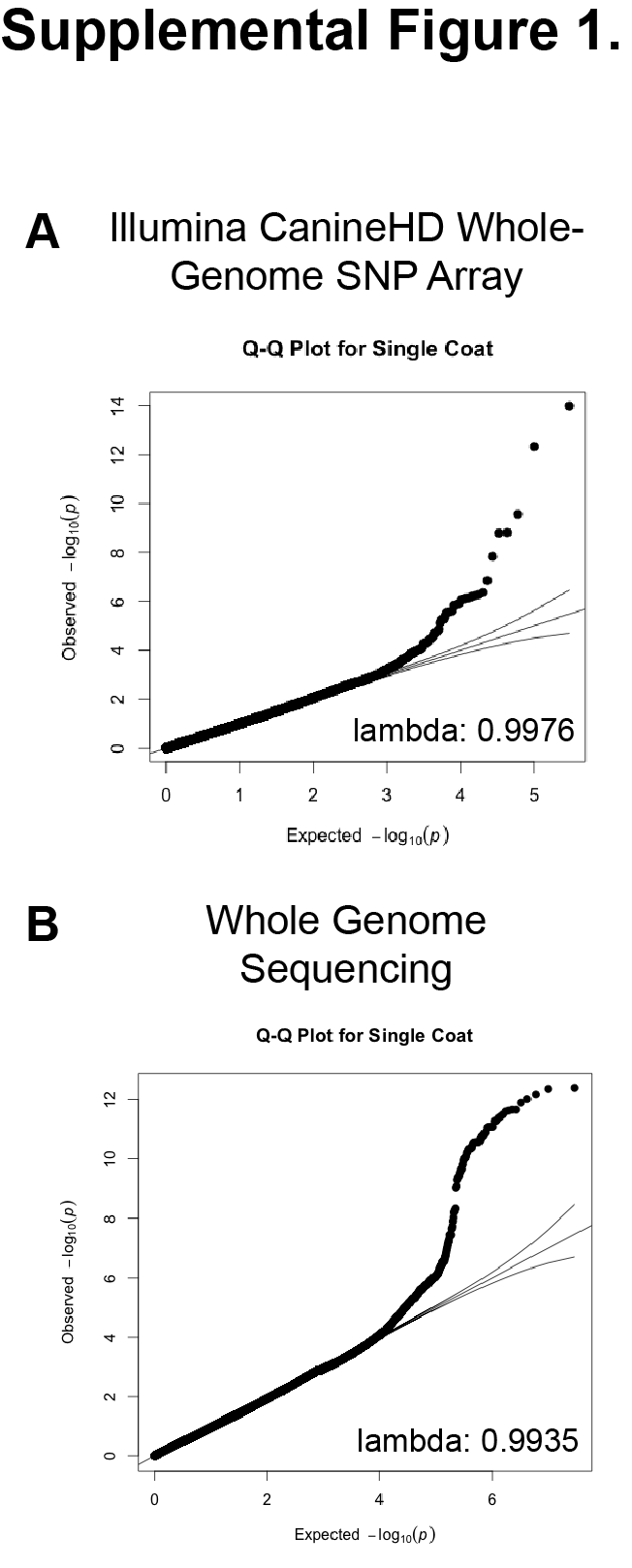

Supplement: Supplementary file 1 [file genes-10-00323-s001.zip › FigureS1.png]

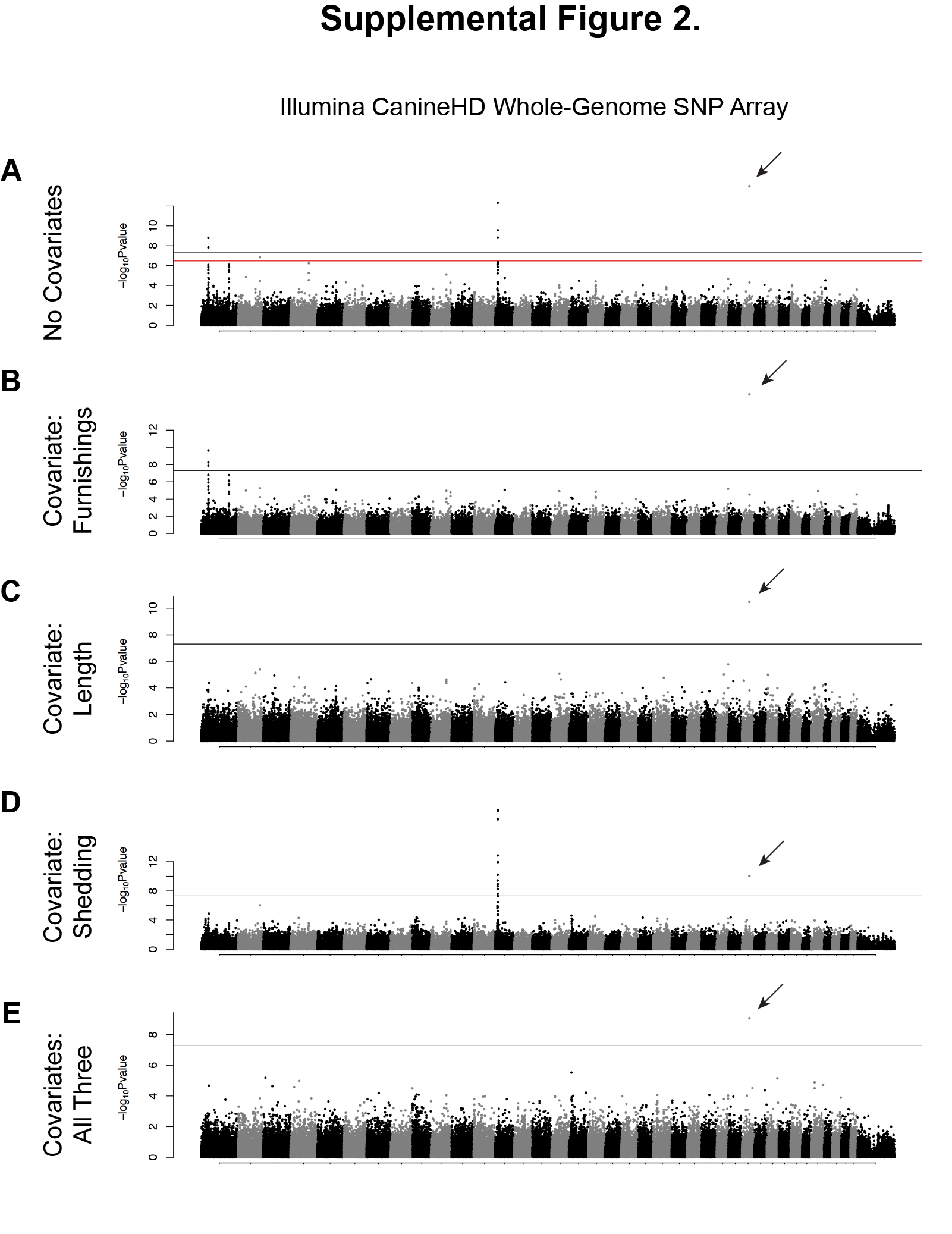

Supplement: Supplementary file 1 [file genes-10-00323-s001.zip › FigureS2.png]
